# Combination of Pyridoxine and Thiamine Treatment in Bilateral Induced Ptosis in a Child: Case Report and Review of Literature

**DOI:** 10.7759/cureus.1573

**Published:** 2017-08-16

**Authors:** Khai-Siang Chai, Mohamad Norsarwany, Ismail Shatriah

**Affiliations:** 1 Department of Ophthalmology, School of Medical Sciences, Universiti Sains Malaysia, 16150 Kubang Kerian, Kelantan, Malaysia; 2 Department of Paediatrics, School of Medical Sciences, Universiti Sains Malaysia, 16150 Kubang Kerian, Kelantan, Malaysia

**Keywords:** bilateral ptosis, vincristine chemotherapy, pyridoxine, thiamine

## Abstract

Ptosis is a rare side effect of vincristine chemotherapy in patients treated for cancer. We report a case of a child with common B-cell acute lymphoblastic leukemia who developed bilateral moderate ptosis following the chemotherapy protocol of the United Kingdom Acute Lymphoblastic Leukemia (ALL) regimen A. The patient showed dramatic clinical improvement after a combination of oral pyridoxine and thiamine treatment. We provide a literature review of this uncommon presentation.

## Introduction

Vincristine is a chemotherapy agent used in pediatric patients with leukemia, lymphosarcoma, Hodgkin's disease, Wilms’ tumour, Langerhans cells histiocytosis (LCH), reticulum cell sarcoma, and neuroblastoma. Neurotoxicity is a known side effect of vincristine. Partial ophthalmoplegia, cranial nerve palsies, and mental depression are common neurological side effects of vincristine. Ptosis is an uncommon side effect. We report the case of a young child who was diagnosed with common B-cell acute lymphoblastic leukemia and developed bilateral moderate ptosis following a chemotherapy regime. The condition was treated successfully with a combination of pyridoxine and thiamine treatment.

## Case presentation

**Figure 1:**
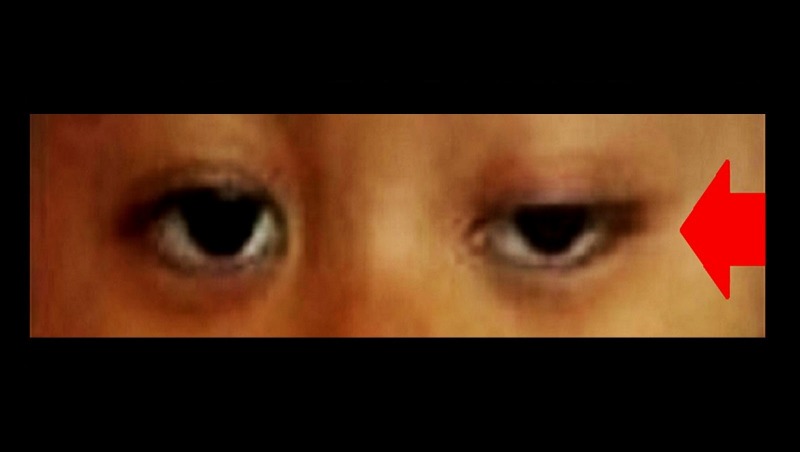
Vincristine-induced bilateral partial ptosis during initial presentation

A two-year-old child with underlying common B-cell acute lymphoblastic leukemia presented with a gradual progressive onset of bilateral drooping of the eyelids after receiving chemotherapy for ten weeks (Figure 1). The ptosis was worse in the left eye compared to the right. Prior to the presentation, she had been receiving the United Kingdom Acute Lymphoblastic Leukemia (ALL) regimen A protocol chemotherapy. This included oral dexamethasone (6.5 mg/m^2^/day), intramuscular L-asparaginase (6000U/m^2^), intravenous vincristine (1.5 mg/m^2^), intrathecal arabinosylcytosine (50 mg) and intrathecal methotrexate (10 mg) therapy. She completed a total of five doses of vincristine, which is equivalent to a cumulative dose of 3.5 mg vincristine (0.36 mg/kg), during the four weeks of the induction phase followed by six weeks of the consolidation phase of chemotherapy.

Her mother noticed that the child had difficulty reading and was frequently rubbing her eyes. The severity of ptosis was constant, and not aggravated by prolonged near activity or time of day. There was no preceding history of recent head or ocular trauma and insect bites. Both parents did not observe apparent ptosis or squint since birth. There was no history of inherited neuropathy or intake of any neurotoxic medication prior to commencing chemotherapy.

The child was able to pick up small objects rapidly without any difficulties and was able to follow objects with a steady gaze. On inspection, there was the presence of upper lid creases in both eyes. Bilateral moderate ptosis was noted, with the right eye upper lid at the upper pupillary margin while the left upper lid was already covering the visual axis. There was no abnormal head posture, eyelids scars, mass, squint, or enophthalmos. The relative afferent pupillary defect was negative. No anisocoria was observed. There was a full range of extraocular movement in both the eyes. Fatigability and Cogan lid twitch tests were negative. Marcus Gunn jaw winking sign was not demonstrable. Other examinations were within normal.

The vital signs were normal. Abdomen examination confirmed hepatosplenomegaly. There were no palpable lymph nodes. Neurological findings like deep tendon reflexes and sensibility were normal. Other cranial nerves examination was unremarkable.

**Figure 2:**
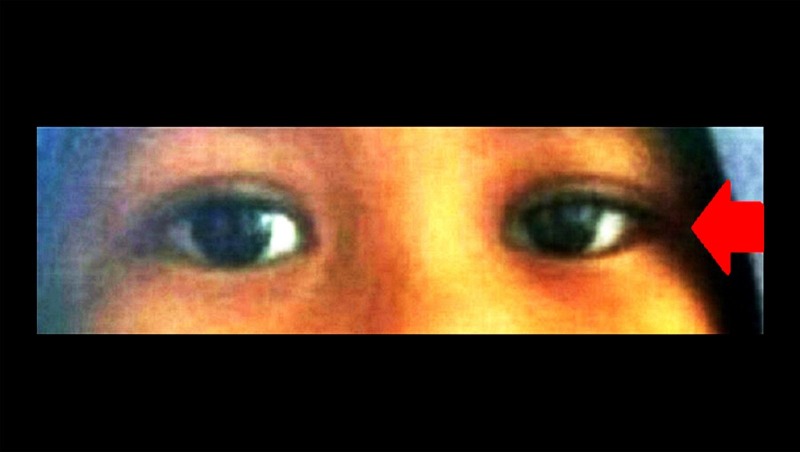
Significant bilateral ptosis improvement after a one-week initiation of pyridoxine and thiamine treatment

**Figure 3:**
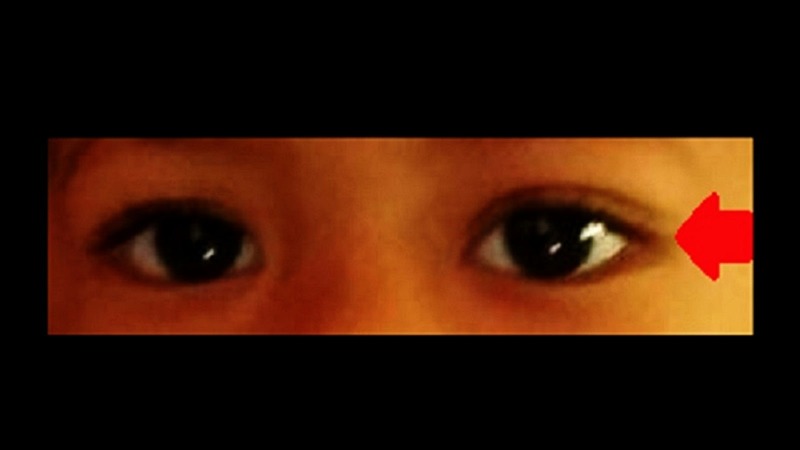
Complete resolution of bilateral ptosis following pyridoxine and thiamine treatment

The patient was treated with syrup gabapentin 100 mg twice a day, and subsequently increased to three times daily, syrup folic acid 0.25 mg once a day and one tablet of neurobion which consists of 200 mg pyridoxine hydrochloride (10 mg/kg), 100 mg thiamine disulfide (5 mg/kg), and 200 mcg cyanocobalamin (10 mg/kg) once a day. The treatment was commenced for a one-month duration. Despite the continuation of chemotherapy, bilateral ptosis markedly improved after one week of the combination of pyridoxine and thiamine treatment, and completely recovered after one month (Figures 2-3). During subsequent follow-up, there was no residual ptosis or recurrence observed.

## Discussion

Peripheral neuropathy is observed when the cumulative dose of vincristine exceeds 6 mg/m^2^. The pathogenesis of vincristine-induced neuropathy is explained by vincristine which prevents microtubules and separates the chromosome pairs during mitosis. This reduces the rate of cell divisions thereby inhibiting tumour growth. Meanwhile, this causes the microtubules to lose function in the maintenance of cell shape and scaffolding, causing axonopathy through axonal transport impairment, and eventually leading to axonal degeneration and death.

Ptosis induced by vincristine post-chemotherapy is an uncommon clinical situation. Table 1 summarizes published cases of the side effects of vincristine in the literature since 2008 until 2017 including our case. The average age of patients who developed ptosis while on vincristine treatment developed ptosis between the ages of 2-11 years. Four patients developed unilateral ptosis. Three of them resolved following pyridoxine and pyridostigmine treatment [1-3]. Including our case, four patients were reported to develop bilateral ptosis alone without other neuropathy [4-6].

**Table 1:** Published vincristine-induced ptosis cases in children from 2008-2017 ALL BFM, Acute Lymphoblastic Leukemia Berlin-Frankfurt-Munster; GPOH-HD, German Society of Pediatric Oncology and Hematology Hodgkin Lymphoma Trial; ALL IC-BFM, Acute Lymphoblastic Leukemia Intensive Chemotherapy-Berlin-Frankfurt-Munster; LCH TRAIL, Langerhans Cells Histiocytosis Tumor Necrotizing Factor Related Apoptosis Inducing Ligand

Author	Year of Publication	Age / Gender	Predisposing Illness	Protocol	Side Effects of Vincristine	Treatment	Final outcome
Gursel, et al. [10]	2009	4 / Female	B cell ALL	ALL BFM-95	Unilateral ptosis	Observation	Improved completely after six weeks cessation of therapy
Dejan, et al. [1]	2009	5 / Male	Common acute lymphoid leukemia	ALL BFM-2002	Unilateral ptosis	Pyridoxine (150 mg/m^2^) & pyridostigmine (3 mg/kg) orally twice daily	Ptosis markedly improved after two weeks treatment and completely resolved after one month
Bhat, et al. [7]	2012	2 / Female	Acute lymphoblastic leukemia	MCP 841	Bilateral ptosis, ophthalmoplegia & polyneuropathy	Same as above	Polyneuropathy and ptosis markedly improved after two weeks treatment & completely resolved after one month
Akbayram, et al. [2]	2013	3 / Female	Stage IIa Wilms’ tumour	EE-4A	Unilateral ptosis	Same as above	Ptosis markedly improved after two weeks treatment & completely resolved after one month
Pandey, et al. [5]	2013	3 / Female	Stage III Wilms’ tumour	DD-4A	Bilateral ptosis	Same as above	Ptosis markedly improved after ten days treatment completely resolved after three weeks
Olcaysu, et al. [8]	2014	11 / Male	Acute lymphoblastic leukemia	GPOH-HD 95	Bilateral ptosis & external ophthalmoplegia	Same as above	Ptosis & ophthalmoplegia markedly improved after ten days treatment & completely resolved after three weeks
Talebian, et al. [6]	2014	2.5 / Male	Wilms’ tumour	EE-4A	Bilateral ptosis	Same as above	Ptosis completely resolved one week treatment
Hatzipantelis, et al. [4]	2015	6 / Male	Acute lymphoblastic leukemia	ALL IC-BFM 2009	Bilateral ptosis	Pyridoxine 200 mg once daily & thiamine 100 mg once daily	Ptosis completely resolved after one month treatment
Karaman, et al. [3]	2016	2 / Male	Langerhans cells histiocytosis	LCH TRAIL	Unilateral ptosis	Pyridoxine (150 mg/m^2^) & pyridostigmine (3 mg/kg) orally twice daily	Ptosis markedly improved after two weeks treatment & completely resolved after three weeks

Four cases showed isolated bilateral ptosis or were associated with polyneuropathy. They markedly improved following pyridoxine and pyridostigmine intake, whereas one case showed almost equal improvement following pyridoxine and thiamine treatment [4-8]. All were reported to tolerate the treatment and did not develop any adverse side effect.

Similiar to Hatzipantelis, et al., our patient had bilateral ptosis post the vincristine treatment and showed significant improvement after neurobion intake which consists of pyridoxine and thiamine. The other cases were treated with a combination of pyridoxine and pyridostigmine [5-6]. The combinations are safe and effective treatment options in vincristine-induced ptosis and they speed up the recovery. The decision of initiating treatment or to be conservative in the condition of mild ptosis remains controversial.

Pyridoxine is an essential cofactor for neuronal protein and neurotransmitter synthesis. The mechanism of action for pyridoxine on peripheral nerves is not clearly understood, but it is known to be involved in numerous biochemical pathways of neural function, including neurotransmitter synthesis, amino acid metabolism, and sphingolipid biosynthesis and degradation.

Thiamine plays a role in nerve membrane conduction, neurotransmitter synthesis, and mitochondrial energy production. In nerve membrane conduction, thiamine triphosphate is thought to activate ion transport, in particular, chlorine but also participation in nerve impulse transmission via sodium channel regulation and release of acetylcholine. It has been reported that thiamine administration suppresses thermal hyperalgesia by reducing hyperexcitability and lessening alterations of sodium currents in injured dorsal root ganglion neurons in rats [9]. Therefore, thiamine administration can help in nerve axons regeneration, reduce pain and aid in balancing sodium currents.

Pyridostigmine is a synthetic quaternary ammonium agent which increases the concentration of endogenous acetylcholine by inhibiting acetylcholinesterase in the neuromuscular junction which in turn increases muscle strength [9]. Pyridostigmine has been used for vincristine-related neuropathy as a reduction of gastrointestinal motility, which is one of the major symptoms commonly found. However, it should be used with caution in patients with bronchial asthma, chronic obstructive pulmonary disease, bradycardia, and cardiac arrhythmias due to its cholinergic effect. Additional research is warranted to assess the role of thiamine, pyridoxine, and pyridostigmine in vincristine induced neuropathy recovery.

## Conclusions

Ptosis is a rare complication of vincristine chemotherapy. Our patient developed bilateral partial ptosis after a cumulative vincristine dose of 3.5 mg but completely recovered after one month of pyridoxine and thiamine supplementation. The recovery time of using the pyridoxine and thiamine combination compares favourably with the pyridoxine and pyridostigmine combination therapy. Nevertheless, such combination treatments accelerate the recovery of ptosis. The ocular side-effects of vincristine chemotherapy are reversible, and early detection is essential to prevent permanent damage.
